# Contract Design: Risk Management and Evaluation

**DOI:** 10.5334/ijic.3616

**Published:** 2018-01-12

**Authors:** Axel C. Mühlbacher, Volker E. Amelung, Christin Juhnke

**Affiliations:** 1 Health Economics and Healthcare Management, Hochschule Neubrandenburg, Neubrandenburg, DE; 2 Institute of Epidemiology, Social Medicine and Health System Research, Hannover Medical School, Hannover, DE

**Keywords:** contract design, integrated care, risk management, healthcare contracts

## Abstract

**Introduction:**

Effective risk adjustment is an aspect that is more and more given weight on the background of competitive health insurance systems and vital healthcare systems. The risk structure of the providers plays a vital role in Pay for Performance. A prerequisite for optimal incentive-based service models is a (partial) dependence of the agent’s returns on the provider’s gain level. Integrated care systems as well as accountable care organisations (ACOs) in the US and similar concepts in other countries are advocated as an effective method of improving the performance of healthcare systems. These systems outline a payment and care delivery model that intends to tie provider reimbursements to predefined quality metrics. By this the total costs of care shall be reduced.

**Methods:**

Little is known about the contractual design and the main challenges of delegating “accountability” to these new kinds of organisations and/or contracts. The costs of market utilisation are highly relevant for the conception of healthcare contracts; furthermore information asymmetries and contract-specific investments are an obstacle to the efficient operation of ACOs. A comprehensive literature review on methods of designing contracts in Integrated Care was conducted. The research question in this article focuses on how reimbursement strategies, evaluation of measures and methods of risk adjustment can best be integrated in healthcare contracting.

**Results:**

Each integrated care contract includes challenges for both payers and providers without having sufficient empirical data on both sides. These challenges are clinical, administrative or financial nature. Risk adjusted contracts ensure that the reimbursement roughly matches the true costs resulting from the morbidity of a population. If reimbursement of care provider corresponds to the actual expenses for an individual/population the problem of risk selection is greatly reduced. The currently used methods of risk adjustment have widely differing model and forecast accuracy. For this reason, it is necessary to clearly regulate the method of risk adjustment in the integrated care contract.

**Conclusions and discussion:**

The series of three articles on contract design has shown that coordination and motivation problems in designing healthcare contracts cannot be solved at no-costs. Moreover, it became clear, that complete contracts in healthcare are unrealistic and that contracts do always include certain uncertainties. These are based on the risk of random, and no contracting party can control these risks completely. It is also not possible to fully integrate these risks in the contract or to eliminate these risks by the parties.

## Background and Objective: How to manage risks and how to evaluate healthcare contracts?

The research questions in this series of three articles focus on how reimbursement strategies (especially Pay for Performance P4P), evaluation of measures and methods of risk adjustment can best be integrated in healthcare contracting. This last article of the series will spot light on the specific topic of risk adjustment as well as evaluation and finally controlling of risks as well as other contractual issues.

Effective risk adjustment is an aspect that is more and more given weight on the background of competitive health insurance systems and vital healthcare systems [1].

The objective of risk adjustment is to generate and provide information about the risks of morbidity and the risk factors within a specific population group. Based on the obtained risk structure the expected utilisation as well as its costs in future periods shall be predicted.

The risk structure of the providers plays a vital role in Pay for Performance (P4P). A prerequisite for optimal incentive-based service models is a (partial) dependence of the agent’s returns on the provider’s gain level.

The risk presented by the population base within a population and indicator oriented contracting must be measured and risk adjustment conducted. Extant risk-sharing modalities will be assessed and their suitability for the different regions and baseline populations.

The aim of using morbidity-oriented classification models of risk adjustment is to achieve a reliable and robust quantification of the expected (present or future) resource utilisation. In terms of their selectivity, these methods are far superior to the “primitive” methods which are based only on the demographic characteristics of age and gender and possibly the status of reduced earning capacity [2].

## Risk Management in healthcare delivery systems

### Risk analysis: Types of risk in healthcare contracts

A transaction between a payer and an integrated care network, like ACOs, is based upon the transfer of property rights. It is only through this transfer that a healthcare reimbursement contract is made possible.

#### Concretization of transaction costs

The healthcare system is organized into work divisions and is based upon segmentation into submarkets. There are a number of exchange relations between payers and service providers which are the starting point for transaction-theory considerations. Central to this is the transfer of property rights, i.e. conclusion of a healthcare reimbursement contract. The specific service content can be concretized according to transaction costs theory by means of specificity, uncertainty and frequency [3]. Even these criteria’s are very hard or almost impossible to measure the framework add significant value to analyses and design contractual relationships [4].

#### Specificity

Location or region is an essential distinguishing feature. The conclusion of a healthcare reimbursement contract in a metropolitan area requires different strategies and different measures of operative implementation than conclusion of such a contract in a rural, perhaps hard to serve, area. Due to the specialism in medical care, an essential feature is human capital. Indication-specific healthcare reimbursement contracts with a high level of specialization require different staffing conditions to, for instance, a full care or case management reimbursement contract. Time specificity plays also an important role in healthcare since the availability (provision and utilisation of options) of services has a considerable influence on the cost and value of the service. Basically, all the characteristics stated could be considered potential specificity characteristics if they render the healthcare service distinguishable for the health insurer or the patient (or other market participants) [4].

#### Uncertainty

Another characteristic of a transaction is the expected level of uncertainty. Events which are outside the control of the contracting parties can affect the transaction. The entrepreneurial risks arising from a healthcare reimbursement contract make it impossible to establish the nature or extent of the services. Ex-ante determination of price-quantity ratio is extremely difficult. Risks relating to population and services as well as future environmental factors cannot be fully accounted for. The services of integrated care cannot only focus on clinical tasks at the given legal issues, but the risk that is taken by the care providers from health insurance when signing the care contract must be taken into special account. First, the risks of care providers in fee for cases- contracts need to be identified. The risk types can be classified as follows [5,6,7]:

- *Risks of random fluctuation:* The actual costs of healthcare provision are in excess of the calculated costs due to random effects. The reason for this is uncertainty with regard to future events.
  - The *incidence risk* is the risk that the number of actually diagnosed cases will exceed the number of cases calculated and expected as a result of random influences (e.g., epidemics such as SARS) [8].
- *Parameters risk:* The actual costs of healthcare exceed the calculated costs due to miscalculation. The reasons for this may be that either the quantities or prices were set too low.
  - The *prevalence risk* describes the risk that the population of fund members contains a higher number of patients with a specific disease than expected. This risk is excluded if in the case of specific indication contracts reimbursement is only made for persons already suffering from the condition.
  - The *severity risk* results from the fact that patients are in a later stage of disease than expected. Fund members then need more intensive intervention and their treatment requires a greater and more cost-intensive level of care.
  - The *duration of stay risk* exists if therapies take longer than expected.
  - There is also a *costs risk* if the cost increases as a result of production factors and services are higher than expected. Necessary drugs, medicines and medical aids must be obtained and additional services are purchased from external service providers. If prices rise unexpectedly to this procurement markets, this can lead to losses [8].
- *Risks of change*: The actual costs of healthcare are in excess of the costs originally calculated following a change in the cost basis assumed. A new and very expensive cancer drug on the market could change all the basic calculations. The more medicine is specialized (and mainly cancer treatments) the more the cost development is unpredictable.
  - The *intensity risk* involves the risk of a patient suffering unforeseen complications or that care is given under more difficult circumstances than expected, resulting in more cost-intensive healthcare procedures.
  - The *guidelines risk* results from the fact that applicable treatment guidelines recommend more intensive or more expensive forms of treatment after a certain time.
  - The *behaviour risk* must be considered if the registered fund members tend towards opportunistic behaviour or if a risk of increased demand without significant increased benefit is to be expected [8].

These risks cannot be considered independently of each other – they are correlated and are mutually dependent. The loss is determined by the probability of actual healthcare costs exceeding the calculated costs. Other general business risks are not negligible (e.g., accidents or fire damage, untimely or incomplete payments by payers or private patients). The impact of these risks is also dependent on the size of the insured population to be served, smaller insured populations are particularly affected by random variances of the cost. The internal compensation, i.e. the compensation of risks is more difficult for smaller groups than for large insurance populations. To reduce the likelihood of negative economic effects and to allow for the achievement of clinical and economic objectives, effective risk management is necessary [5]. The composition and risk structure of the insured population have a crucial impact on the actuarial risk. These risks require to choose the right pricing model for the adequate determination of the amount of capitation or other fees [7,9].

#### Frequency

The frequency with which a transaction is carried out has an effect on the introduction of a control and monitoring system. A specialized control and monitoring system is generally only worthwhile for frequent and highly specific transactions. Frequent transactions which can be settled on the market without difficulty do not necessitate the introduction of a control and monitoring system.

### Risk Management within healthcare contracts

Reimbursement with a high degree of generalization requires more awareness of the morbidity structure, according to the assumption that this is consistent with the treatment costs. The morbidity orientation serves as a determinant of the appropriate level of reimbursement and reward. It depends on the accuracy of the calculation: when forecasting healthcare costs of insured clients often only average cost of care are considered the heterogeneous utilisation or risk of morbidity in the population structure are not taken into account. A result-oriented remuneration and reimbursement only makes sense if it can be weighted according to the population-side requirements [8].

Risk adjusted contracts ensure that the reimbursement roughly matches the true costs resulting from the morbidity of a population. If reimbursement of care provider corresponds to the actual expenses for an individual/population the problem of risk selection is greatly reduced. An increase of the profit margin can therefore be achieved only through effective healthcare delivery, incentives to reduction in quality might occur. An avoidance of risk selection is achieved if care providers are adequately reimbursed for severe cases or high risk patients. However, there is an incentive to increase the profit share as a result of efficient care delivery [8].

The currently used methods of risk adjustment have widely differing model and forecast accuracy. For this reason, it is necessary to clearly regulate the method of risk adjustment in the contract [10,11]. A risk-adjusted fee is a compromise between efficient risk allocation and efficient incentives for action in integrated care [12].

Integrated care networks as well as ACOs are also likely to bear financial risk, receiving greater payments for the care of chronically ill patients and accepting at least partial responsibility for the costs of specialists’ visits, tests, emergency room visits, and hospitalizations. Since the containment of insurance risks is not one of the core competences of service providers, risks which are not the responsibility of the service providers should be excluded. But we have to bear in mind that in healthcare the separation of responsibility is difficult. The main reasons are the role of the patient and unpredictability. Risk-adjusted contracts should ensure that reimbursement is approximately equal to the actual costs resulting from the morbidity of a population. The basic principle of risk adjustment lies in identification and weighting of morbidity criteria and risk factors relevant to prognosis [10]. The risk factors can be identified on the basis of various sources of information: demographic data (age and gender), patient data on services utilised, survey data from member surveys and diagnoses from the service performance data. Using these basic prognosis parameters for a particular year, attempts are made to predict the services which will be utilised or the healthcare costs for future periods. Avoidance of risk selection is achieved by ensuring that service providers also receive appropriate remuneration for severe cases or high risks. If the reimbursement received by service providers is equivalent to the actual costs for an individual or population, then the problem of risk selection is considerably reduced. Risk classification procedures are applied in various areas:

- for calculation of risk-adjusted per capita rates for group insurance offers,
- for standardisation of risk for comparisons of feasibility or quality comparisons (provider profiling) and
- for the reimbursement of service providers.

Closely related to the risk adjustment topic is the question of the minimum number of patients enrolled in ACOs. The smaller the numbers of patients the more import are sophisticated methods of risk adjustments. The estimated minimum number of enrolees is between 100.000–150.000.

**Figure 1:**
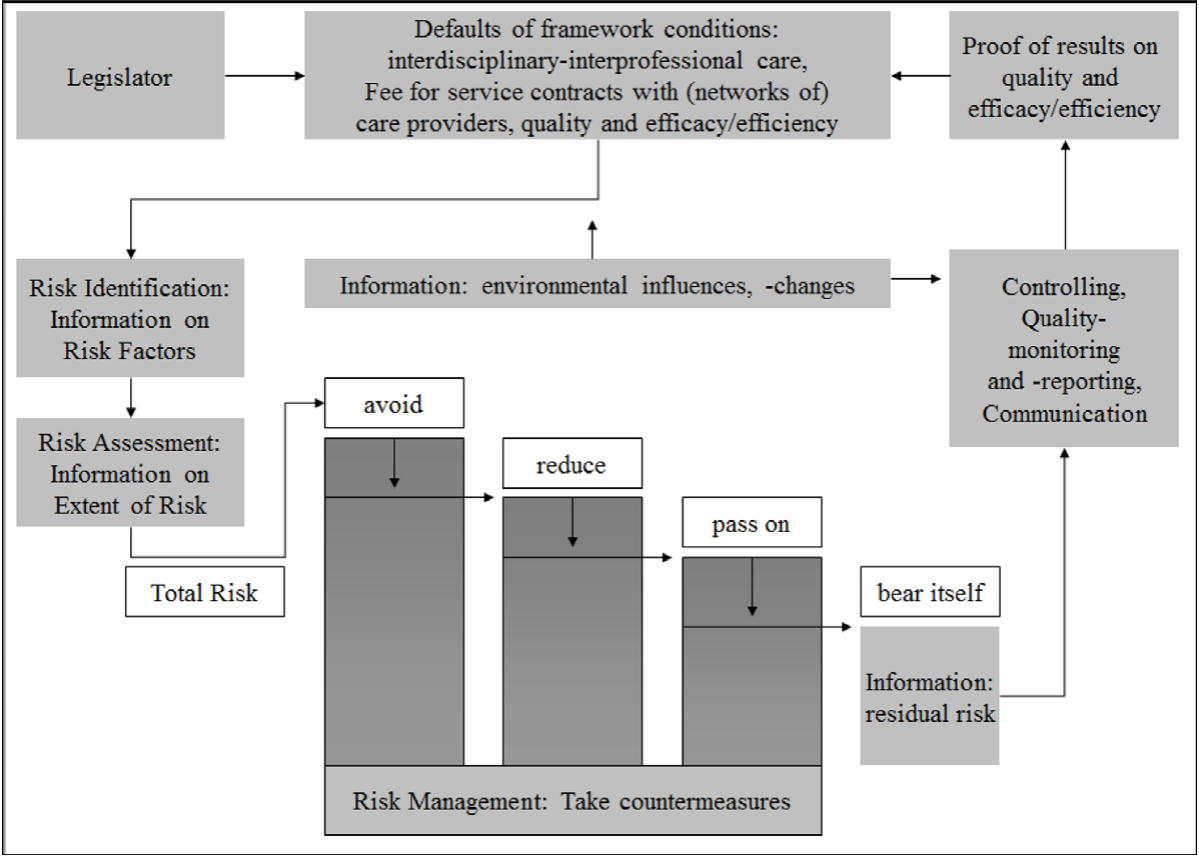
Risk Management in Healthcare Contracts (own figure).

The design of contracts for integrated care programs should be geared to the needs of the Parties (see Figure 1). Contracts should concretize which care functions are performed by the care providers and on the other hand the contract offers the opportunity to flesh out the extent to which the insurance function is transferred from the payers to care providers. Exceeds the risk from the care contract a certain limit, the healthcare provider (as well as the payers) should refrain from the business. The unique design of the care contracts offers the opportunity to clearly recognize the risk and to avoid the risks. Based on the contractual features of integrated care, the level of capitation, including the risk premium will be determined. The contracts for integrated care can include agreements to avoid uncertainties [11,13].

## Controlling and evaluation of healthcare contracts

### Indicators and Benchmarks

The evidence-based medicine (EBM) is an important aspect in the control of healthcare (delivery) risks. Standardized medical reviews, standardized economic reviews and evidence-based guidelines can be the basis for the evaluation of care processes.

The comparison of the actual results (actuals) to the desired outcomes (plan or target numbers), requires a comprehensive information management with respect to the clinical, administrative and financial processes in care delivery in the integrated care (for further details: Wrightson, 2002) [14]. Important parts of risk management are metrics that demonstrate the successful implementation of care processes. These indicators (“performance indicators”) or comparative figures (“benchmarks”) are the basis for the review of the success of a care contract. They allow a comparison of the actual results (actuals) to the desired outcomes (plan or target numbers) [15]. This information should reflect the clinical, financial and administrative requirements:

- Composition of the insured clientele by age, gender, social index and morbidity.
- Percentage of the population, which was reached through agreed special services (e.g., through early diagnosis measures, screenings).
- Utilisation of key performance areas, particularly those sectors whose utilisation should be reduced in case of success.
- Expenses/costs of care (e.g., cost per treated patient, according to the type and severity of illness).
- Efficiency of administrative operations (cost per patient or every €, $).
- Documentation of the quality of clinical services using comparable quality indicators.

The key to successful risk management in healthcare is the knowledge of the administrative and clinical processes. Only by understanding the dynamic processes of care, the management is able to make appropriate decisions. This requires a comprehensive information management across the clinical, administrative and financial processes in care delivery within integrated care networks (for further comments: Wrightson 2002). Information management can be supported by information from the organisational quality management (quality management), by a gatekeeper (gatekeeping) or Case Manager (Case Management) and the Utilisation Management and Review (concurrent review, retrospective review) [11].

### Evaluation: Success of care contracts and healthcare delivery networks

Each integrated care contract includes challenges for both payers and providers without having sufficient empirical data on both sides. These challenges are clinical, administrative or financial nature. Especially the targeted diseases with enormous differing treatment strategies (treatment pathways), high volatility in the utilisation and widely varying costs are significant challenges for these innovative forms of care. Successful care contracts guarantee a need-based and economical healthcare delivery, the satisfaction of the insured and provide adequate access to the services. For most diseases, early detection and timely according to anabolic processes of care are needed. Contracts that do not ensure sufficient access produce poor medical results (outcomes) and cause high administrative costs resulting in image and sales problems for insurers.

Before an assessment of the success can be performed, the success parameters need to be defined. In addition, the contracting parties should analyse the financial and clinical risks of a care program in advance. At a later stage, the success can be measured in terms of control of financial risks, customer satisfaction and compliance with specific quality parameters. In addition to quantitative methods, qualitative methods should be used. Care contracts and programs can be evaluated using the following parameters:

- *Medical Quality:* The medical quality (outcomes) must be increased by the measures and services of integrated care, or at least maintained. The quality measurement can be performed with the aid of quality indicators (performance indicators). These indicators can be used for external comparisons, but can also for internal quality management.
- *Customer satisfaction:* The health insurance, but also the care providers are reliant on the acceptance of the healthcare delivery programs by the insured and also positive rating. It is to check whether the patient has received all the necessary services, how the insured evaluates the care and if he has the opinion that his services were denied. Patient satisfaction is enhanced by a smooth and comprehensive management of care processes. The customer loyalty to the health insurance company is affected by the administrative processes in the processing. The measurement of customer satisfaction is conducted via patient or insured surveys. These surveys can be done regionally or nationwide. A survey at the entry or exit of the insured into the program gives information about which parameters influence the decision to change.
- *Financial Success:* The restructuring of the care processes, new services and the development of new organisational forms require high investments. From the business perspective, it requires the analysis of the returns on investment (ROI), i.e. the used means must be generated by the services offered within the integrated care program. Investments to develop integrated care will not directly lead to income; it is rather a societal perspective to quantify the savings for a particular insured clientele. The measurement approach for financial success can refer to the documentation of individual cases, the development of costs of comparable insured populations or on the resource use for special care processes.
- *Adjusting for risk factors*: In order to prove actual quality, satisfaction and cost effects, it requires the adjustment of the data in terms of risk factors. The differences between care programs or care contracts regarding the use of medical services and the costs can only be documented after an adjustment. Only then the impact of both the risk factors and the care program on the other hand become clear.

This information from the evaluation of an integrated care program can serve as a basis for a result-oriented remuneration. In the interests of patients, the impact of costs and effects of care programs and contract design on the clinical quality of care and patient satisfaction should be evaluated [11].

## Discussion and Conclusion

Each integrated care contract includes challenges for both payers and providers without having sufficient empirical data on both sides. These challenges are clinical, administrative or financial nature.

The series of three articles on contract design has shown that coordination and motivation problems in designing healthcare contracts cannot be solved at no-costs. Moreover, it became clear, that complete contracts in healthcare are unrealistic and that contracts do always include certain uncertainties. These are based on the risk of random, and no contracting party can control these risks completely. It is also not possible to fully integrate these risks in the contract or to eliminate these risks by the parties.

However, it is not advisable to allow an agreement to renegotiate individually for each risk event. It is therefore important to ensure ex ante that the contract clearly states the circumstances (risk events), which are authorized under renegotiation. Moreover, it must be noted, who bears the risks as well as the potential shares of risks, the contracting parties shall bear.
